# Computational Characterization of Pathogenic LMNA Missense Variants: Structural Instability, Altered Binding, and Conformational Dynamics

**DOI:** 10.1155/humu/2444803

**Published:** 2026-08-26

**Authors:** Emre Aktaş, Ceren Nizamoğlu, Salvador Ventura

**Affiliations:** ^1^ Department of Molecular Biology and Genetics, Faculty of Arts and Sciences, Yıldız Technical University, Istanbul, Türkiye, yildiz.edu.tr; ^2^ Institut de Biotecnologia i de Biomedicina and Departament de Bioquímica i Biologia Molecular, Universitat Autònoma de Barcelona, Bellaterra, Barcelona, Spain, uab.cat

**Keywords:** Evo2, intrinsic disorder, Lamin A, laminopathies, loss-of-function variants, molecular docking, molecular dynamics, protein structure, variant prioritization

## Abstract

**Background:**

Mutations in the *LMNA* gene underlie a broad spectrum of laminopathies, including muscular dystrophies, cardiomyopathies, and premature aging syndromes; however, the molecular mechanisms by which missense variants disrupt Lamin A structural integrity remain incompletely characterized. Systematic computational approaches for prioritizing pathogenic variants and elucidating their structural consequences are critically needed.

**Methods:**

An integrated multistep in silico framework was employed to investigate the structural and functional consequences of *LMNA* missense variants. Variant prioritization was performed using the Evo2 nucleotide language model via delta log‐likelihood scoring, followed by bioinformatic annotation using SIFT, PANTHER‐PSEP, PhD‐SNP, and E‐SNPs&GO. Protein stability assessment was conducted with DynaMut, INPS‐MD, I‐Mutant2.0, and MUpro. Variants localized within globular domains—N456D, N456T, and G465D—together with the known pathogenic variant M540T as a positive control, were selected for three‐dimensional structural modeling using PyMOL and AlphaFold2, molecular docking with lonafarnib as a reference ligand via AutoDock Vina, and 100 ns molecular dynamics simulations using GROMACS with the Amber ff14SB force field. Conformational dynamics were characterized through principal component analysis and free‐energy surface construction.

**Results:**

Evo2‐based screening of the full LMNA coding sequence identified 50 high‐priority loss‐of‐function variants, of which N456D, N456T, and G465D were retained for structural investigation based on their globular domain localization and multitool pathogenicity predictions. All three variants were consistently predicted to alter physicochemical properties and reduce structural stability relative to wild‐type Lamin A. Molecular docking revealed mutation‐dependent changes in lonafarnib binding profiles. The known pathogenic control M540T exhibited comparable structural and dynamic behavior, supporting the reliability of the prioritization workflow. Molecular dynamics analyses demonstrated altered RMSD trajectories, increased residue‐level flexibility, and modified hydrogen bonding patterns in mutant systems. Free‐energy landscape analyses revealed expanded conformational basins, particularly pronounced in the G465D variant, indicating increased structural plasticity.

**Conclusion:**

This integrated computational framework provides a systematic strategy for prioritizing pathogenic *LMNA* variants and characterizing their structural consequences at the atomic level. The identified variants—N456D, N456T, and G465D—represent structurally disruptive substitutions consistent with the established role of globular domain destabilization in other laminopathy‐associated variants, offering testable hypotheses for experimental validation in cellular and animal models.

## 1. Introduction

In multicellular organisms, preserving cellular structural integrity is essential for maintaining tissue homeostasis, particularly in mechanically active tissues such as skeletal and cardiac muscle and skin. Mechanical coupling between the cytoskeleton and the nucleus enables efficient transmission of mechanical signals and ensures nuclear stability under continuous mechanical load. Within this mechanotransductive network, the LMNA gene plays a central role by encoding A‐type lamins, which constitute the core structural components of the nuclear lamina [1–3]. Lamin A and Lamin C are essential for maintaining nuclear shape, regulating nuclear stiffness, and preserving chromatin organization [4]. Their mechanical function directly influences cellular resilience to deformation and contributes to tissue‐specific mechanical properties [5, 6]. This role is particularly critical in tissues exposed to repetitive mechanical stress, where impaired nuclear mechanics can lead to progressive cellular damage [7]. In addition to their structural role, A‐type lamins participate in a wide range of nuclear processes, including nuclear envelope disassembly during mitosis, chromatin organization, transcriptional regulation, DNA repair, and telomere maintenance. Through these functions, Lamin A/C plays a pivotal role in maintaining genomic stability and cellular integrity [6–10]. Mutations in the LMNA gene give rise to a heterogeneous group of disorders collectively termed laminopathies, encompassing muscular dystrophies, cardiomyopathies, lipodystrophies, and premature aging syndromes [11–13]. Among these, missense mutations are the most frequently observed variant type and are particularly prevalent in laminopathies affecting mechanically stressed tissues. These substitutions often induce subtle but functionally significant alterations in lamin structure, ultimately compromising nuclear stability and cellular viability. A growing body of evidence suggests that LMNA‐associated pathologies arise not only from structural defects but also from impaired mechanotransduction at the nuclear envelope. According to this model, mutations in A‐type lamins reduce the nucleus′s ability to withstand mechanical stress, leading to nuclear deformation, accumulation of DNA damage, and progressive cellular dysfunction [14]. This vulnerability is especially pronounced in muscle and cardiac tissues, where mechanical forces are continuously applied. At the molecular level, Lamin A is synthesized as Prelamin A and undergoes a tightly regulated series of posttranslational modifications before reaching its mature form. Proper processing of Prelamin A is essential for nuclear lamina assembly and structural integrity [15]. Even minor amino acid substitutions within the Prelamin A sequence can disrupt this maturation process, alter protein–protein interactions, and lead to structural instability [16]. Consequently, detailed investigation of amino acid–level alterations in Lamin A is critical for understanding the molecular basis of LMNA‐related disorders [9, 14, 16].

In this study, we employed an integrated in silico approach to investigate the structural and functional consequences of LMNA variants with potential pathogenic relevance. To systematically explore these effects, we designed a multistep computational workflow consisting of mutation modeling, stability prediction, molecular docking, molecular dynamics (MD) simulations, and principal component analysis (PCA). Following the identification of mutation‐prone regions using language model–based evolutionary analysis, amino acid substitutions corresponding to high‐risk positions were mapped onto the Lamin A sequence. Variants predicted as benign or located outside structurally meaningful regions were excluded, and only substitutions residing within well‐defined globular domains were retained for further investigation. These selected variants were subjected to three‐dimensional (3D) structural modeling to evaluate mutation‐induced conformational changes. To further explore the functional implications of these alterations, molecular docking analyses were performed using lonafarnib as a reference ligand [17], followed by MD simulations to assess the stability and dynamic behavior of the resulting complexes. Finally, PCA and free‐energy surface (FES) calculations were conducted to characterize mutation‐dependent conformational variability and energetic profiles.

## 2. Materials and Methods

### 2.1. Evo2‐Based Variant Prioritization

To prioritize potentially deleterious variants in the LMNA gene, a zero‐shot prediction approach based on the Evo2 (https://github.com/ArcInstitute/evo2/blob/main/notebooks/) nucleotide language model was employed. Evo2 was selected as the initial screening tool because it provides a sequence‐only, zero‐shot scoring approach applicable across variant types, including non‐SNV events such as insertions, deletions, and duplications, for which alignment‐based and missense‐specific predictors such as AlphaMissense and GPN‐MSA cannot generate scores. In benchmarking against ClinVar‐annotated variants, Evo2 has been reported to outperform these and other established methods specifically for non‐SNV coding variants, while remaining competitive (though not superior) for coding SNVs [18]. Evo2 estimates the functional impact of single‐nucleotide substitutions by comparing the log‐likelihood of reference and variant sequences within their genomic context [18]. For each candidate position, a fixed‐length sequence window was extracted, and delta log‐likelihood scores were calculated as the difference between variant and reference probabilities. More negative scores indicate substitutions that deviate strongly from evolutionary constraints and are therefore more likely to be functionally disruptive. Variants were ranked according to their Evo2 delta scores [19], and those predicted as benign or corresponding to duplications were excluded. The remaining high‐confidence variants were mapped to their amino acid substitutions and used for subsequent structural and molecular analyses. This Evo2‐based filtering strategy enabled unbiased prioritization of LMNA variants based on evolutionary and sequence‐level constraints, providing a robust foundation for downstream structural and dynamic investigations. Positions with high delta scores were interpreted as having a greater likelihood of functional or evolutionary impact. Although the full genomic sequence of the LMNA gene was included in the computational analysis, downstream variant prioritization and SNP selection were restricted exclusively to positions located within the protein‐coding regions responsible for LMNA protein synthesis. Only the Top 50 substitutions classified as loss of function (LOF) within the LMNA coding regions were retained for subsequent annotation and interpretation, as these variants are most likely to result in amino acid changes with a substantial impact on LMNA protein structure and function. Here, the term “LOF” is used in a descriptive sense to denote variants predicted by Evo2 to strongly deviate from evolutionary sequence constraints, and does not imply a specific established disease mechanism such as haploinsufficiency; the missense variants examined in this study are more consistent with a dominant‐negative mode of action, as commonly proposed for LMNA missense variants [20].

Variant information corresponding to the most significant substitutions was annotated at both the nucleotide and amino acid levels using public genomic databases including dbSNP (https://www.ncbi.nlm.nih.gov/snp/), ClinVar, gnomAD, and UniProt [21, 22]. The full‐length amino acid sequence of the LMNA protein (UniProt ID: P02545) was also retrieved from the UniProt database in FASTA format to support subsequent bioinformatic analyses.

### 2.2. Prediction of Intrinsically Disordered Regions (IDRs) in the LMNA Protein

Identification of IDRs in proteins is crucial for understanding their structural flexibility and functional dynamics. To predict potential IDRs within the LMNA protein, the CAID (Critical Assessment of Intrinsic Disorder Prediction) platform was utilized [23, 24]. This platform integrates the outputs of multiple state‐of‐the‐art computational predictors to provide a consensus‐based assessment of protein disorder [25]. Through CAID analysis, putative disordered regions were identified based on the aggregated predictions of several bioinformatic tools. The resulting data were downloaded in TSV format and processed using Python to generate a heatmap visualizing the disorder propensity across different predictors. Regions exhibiting either high disorder scores or an increased likelihood of being mutation‐prone were flagged and retained for further analysis in downstream steps.

### 2.3. Prediction of Functional Consequences and Disease Associations

To assess the potential biological relevance of the identified SNPs—particularly their capacity to disrupt the structural or functional integrity of the LMNA protein—a series of in silico tools were employed. In this stage, the candidate positions flagged in the previous analyses (e.g., mutation‐prone or disordered regions) were subjected to computational prediction pipelines designed to evaluate their functional and pathological impact. First, variants predicted to be “damaging” by SIFT (i.e., those with a score of < 0.05), which estimates the effect of amino acid substitutions based on evolutionary conservation, were selected for further analysis [26]. In addition, the PANTHER‐PSEP tool was used to identify SNPs located in evolutionarily conserved regions, classifying them as “possibly damaging” based on preservation time metrics [27]. Furthermore, variants classified as “disease” by the PhD‐SNP tool—an SVM‐based predictor trained to distinguish between disease‐related and neutral mutations—were recorded [28]. Lastly, the E‐SNPs&GO tool, which integrates sequence features, evolutionary profiles, and Gene Ontology (GO) annotations, was used to identify SNPs with a high probability of being disease‐associated [29]. The combined use of multiple complementary in silico tools enhances the reliability of functional predictions by integrating diverse evolutionary, structural, and functional evidence. These tools were specifically selected because they capture complementary and largely independent lines of evidence: SIFT and PANTHER‐PSEP rely primarily on evolutionary sequence conservation, whereas PhD‐SNP and E‐SNPs&GO are machine learning classifiers that additionally incorporate structural and GO‐derived features. Requiring convergence across these methodologically distinct approaches, rather than relying on any single predictor, was intended to reduce the risk of tool‐specific bias in variant prioritization.

### 2.4. Prediction of the Impact on Protein Stability

Variants previously classified as “damaging” or “pathogenic” through functional analyses were further evaluated for their potential impact on protein stability using two distinct in silico tools: DynaMut and INPS‐MD. In the DynaMut analysis, variants with a positive *Δ* *Δ* *G* value, indicating a predicted destabilizing effect on protein structure, were considered for further evaluation [30]. Conversely, the INPS‐MD tool was used to identify variants with a negative *Δ* *Δ* *G* value, also suggestive of decreased structural stability, and these were included in the study [29]. DynaMut and INPS‐MD were selected for this stage because they represent two methodologically distinct approaches to stability prediction: DynaMut employs structure‐based graph theory and normal mode analysis derived from the 3D protein structure, whereas INPS‐MD relies on sequence‐ and profile‐based machine learning independent of structural input. Requiring agreement between a structure‐based and a sequence‐based predictor was intended to provide more robust evidence of destabilization than either method alone.

### 2.5. Prioritization and Clinical Validation of Potentially Pathogenic SNPs

Following protein stability assessments and other in silico evaluations, a subset of high‐risk variants was prioritized for further investigation. In this stage, variants with a negative *Δ* *Δ* *G* value—indicating decreased stability—were selected using I‐Mutant2.0 and MUpro [31]. In addition, variants predicted to be pathogenic with a probability score of ≥ 0.5 by MutPred2 were included [32]. To determine the clinical novelty of these computationally predicted pathogenic variants, the ClinVar database was queried. Variants that were already annotated in ClinVar as disease‐associated were excluded from further consideration, as the primary aim of this study was to identify potentially novel or previously uncharacterized pathogenic SNPs [33]. I‐Mutant2.0 and MUpro were included alongside DynaMut and INPS‐MD as additional, independently trained machine learning predictors of stability change, further reducing reliance on any single algorithm. MutPred2 was selected at this stage because, unlike the stability‐focused tools above, it integrates structural, functional, and evolutionary features into a single composite pathogenicity probability score, providing a complementary, disease‐relevance‐oriented line of evidence rather than a stability‐specific one.

### 2.6. Prediction of Wild‐Type and Mutant LMNA Amino Acid Properties

To assess the potential structural and functional effects of candidate mutations identified in previous analyses, the HOPE (Have Our Protein Explained) tool was used [34]. This tool evaluates the impact of amino acid substitutions by analyzing changes in key physicochemical properties such as size, charge, and hydrophobicity [34]. Through this analysis, the possible consequences of mutations on the LMNA protein′s overall conformation and molecular interactions were inferred.

### 2.7. Estimation of Physicochemical Properties of Candidate Amino Acid Positions and Their Native Residues

The physicochemical properties of both the wild‐type and mutant forms of the LMNA protein were computed using the ExPASy ProtParam tool [35]. Key parameters—including molecular weight, theoretical isoelectric point (pI), number of charged amino acids, instability index (II), aliphatic index, and GRAVY (grand average of hydropathicity)—were analyzed and compared between the wild‐type and mutant sequences to assess potential changes in protein behavior and stability [35].

### 2.8. Structural Modeling and Docking Preparation of Wild‐Type and Mutant LMNA Proteins

To investigate the structural effects of candidate mutations and assess their potential interaction with small‐molecule ligands, 3D structural models of the LMNA protein were generated and evaluated. The crystal structure of the LMNA rod domain (PDB ID: 1IFR) was used as a reference for the wild‐type conformation. Site‐directed mutant models corresponding to prioritized variants were constructed using PyMOL by introducing single amino acid substitutions into the wild‐type structure [36]. Additionally, full‐length protein models were predicted using AlphaFold2, and only those with high per‐residue confidence scores (pLDDT ≥ 80) were retained [37]. To ensure biological relevance and structural interpretability, only mutations located within globular domains of the LMNA protein were included in the docking stage. Variants situated in predicted nonglobular or IDRs were excluded, as docking simulations in such flexible, unresolved regions are generally considered unreliable and may lead to artifacts in ligand‐binding predictions [38, 39]. Based on these criteria, G465D, N456T, and N456D were selected as the final candidate mutations, as they reside within the structured (globular) regions of LMNA. Separate 3D models were generated for each mutant structure using both PyMOL and AlphaFold‐based modeling workflows. The mutant and wild‐type structures were structurally aligned to assess mutation‐induced conformational changes. All models underwent energy minimization in a Linux environment using UCSF Chimera 1.19 to optimize local geometry and improve structural accuracy [40]. For docking analysis, lonafarnib (PubChem CID: 148195), a clinically approved farnesyltransferase inhibitor used in the treatment of Hutchinson–Gilford progeria syndrome, was selected as a reference ligand due to its clinical relevance in laminopathy‐related pathologies [17]. The ligand structure was retrieved from PubChem and subsequently energy‐minimized using UCSF Chimera 1.19. The receptor and ligand files were then prepared for docking by assigning charges, adding hydrogens, and removing nonessential heteroatoms, ensuring compatibility with downstream molecular docking tools. It is important to emphasize that lonafarnib is not known to directly interact with Lamin A under physiological conditions, as its primary mechanism of action involves inhibition of farnesyltransferase [41]. Therefore, in the present study, lonafarnib was not used to model a biologically validated LMNA–ligand interaction. Instead, it was selected as a clinically relevant and structurally well‐characterized reference compound to probe mutation‐induced changes in the physicochemical environment of the LMNA globular domain. Within this framework, molecular docking and subsequent MD simulations were applied as comparative computational tools to assess how specific amino acid substitutions influence local interaction patterns, conformational stability, and dynamic behavior of the protein structure.

### 2.9. Molecular Docking of Wild‐Type and Mutant LMNA Proteins With Lonafarnib Using Blind and Flexible Approaches

Molecular docking analyses were performed to explore potential binding modes between lonafarnib (PubChem CID: 148195) and both wild‐type and mutant (G465D, N456T, and N456D) forms of the LMNA protein, using energy‐minimized structures obtained from prior modeling steps. As a positive control, the known pathogenic variant M540T (rs267607547; ClinVar: Hutchinson–Gilford progeria syndrome) was docked with lonafarnib using the identical protocol; it was the only ClinVar‐pathogenic control that localizes to the C‐terminal Ig‐fold globular domain, whereas the remaining controls map to the *α*‐helical rod domain and therefore lack a defined docking pocket (Supporting Information 1: Table S1). Although lonafarnib primarily targets farnesyltransferase, its potential interactions with the LMNA globular domain were investigated here as a hypothesis‐generating computational approach [17]. Docking was performed with AutoDock Vina integrated into UCSF Chimera 1.19 on a Linux platform. Two complementary approaches were applied: blind docking, which scanned the entire protein surface without predefined binding site constraints, and flexible docking, which targeted a known binding region while allowing side‐chain flexibility around the pocket [42, 43]. These dual strategies were used to enhance the reliability of predictions by capturing both global binding potential and local conformational adaptability. Only mutations located in globular regions of LMNA were considered, as docking within nonglobular or disordered regions is prone to unreliable outcomes. Following docking, binding affinity scores were evaluated, and complexes showing favorable interactions were selected for subsequent MD simulations.

### 2.10. MD Simulations

MD simulations were performed to assess the structural stability and dynamic behavior of LMNA–lonafarnib complexes, including both the wild‐type and mutant variants (G465D, N456T, and N456D), which were selected based on favorable molecular docking results [44]. Simulations were conducted using the GROMACS package, with the Amber ff14SB force field employed for accurate representation of protein–ligand interactions [45]. Each complex was solvated in an SPC (simple point charge) water model within a triclinic simulation box, ensuring adequate buffer distance from the protein surface. The systems were neutralized with Na^+^ and Cl^-^ ions to achieve a physiological salt concentration of 0.15 M, mimicking cellular ionic conditions [46]. Energy minimization was performed using the steepest descent algorithm for 5000 steps to eliminate steric clashes and optimize the initial geometry. This was followed by equilibration under NVT and NPT ensembles at 300 K and 1.0 bar, each run for 300 ps, to stabilize temperature and pressure prior to production. Production MD simulations were carried out for 100 ns using the leapfrog integrator, and trajectory snapshots were saved every 20 ps, yielding 5000 frames per simulation. Postsimulation analyses were performed to evaluate the dynamic and structural features of each complex. Specifically, root mean square deviation (RMSD) was calculated to monitor the overall structural stability throughout the simulation, whereas root mean square fluctuation (RMSF) was used to assess local residue‐level flexibility and potential mutation‐induced mobility shifts. Solvent accessible surface area (SASA) was analyzed to examine changes in protein compactness and solvent exposure, and hydrogen bond (H‐bond) analysis was conducted to quantify key stabilizing interactions between the protein and ligand [44, 46–48].

#### 2.10.1. PCA and FES Construction

To further investigate the conformational dynamics of the LMNA–lonafarnib complexes during the MD simulations, PCA was performed using GROMACS 2023.2 [49]. Backbone atomic fluctuations were used to construct the covariance matrix via the *gmx covar* tool, followed by eigenvector decomposition to extract the dominant modes of motion. The first two principal components (PC1 and PC2) were selected, and trajectory projections were computed using *gmx anaeig*. To explore the conformational landscape, a two‐dimensional FES plot was generated based on the projections of PC1 and PC2 [49, 50]. FES construction was carried out by applying Boltzmann inversion to the normalized 2D histogram of principal component coordinates [50]. The M540T positive control complex was simulated under the same 100 ns protocol and analyzed with the identical pipeline (RMSD, RMSF, radius of gyration (Rg), H‐bond count, PCA, and FES) used for the wild‐type and candidate‐variant systems. All visualizations were implemented in *Python* (V3.11) using *Matplotlib* and *NumPy*. This analysis provided insights into the conformational diversity, dominant motions, and free‐energy basins sampled by the protein–ligand complexes, thereby complementing the structural and thermodynamic findings derived from RMSD, RMSF, SASA, and hydrogen bonding analyses.

## 3. Results

### 3.1. Identification and Filtering of LOF Variants in the Protein‐Coding Regions of LMNA

Initially, the Top 50 variants with the most negative Evo2 delta scores were selected as potential LOF candidates in the LMNA gene (Table 1). These variants represent positions predicted to have the highest functional impact based on evolutionary constraint inferred by the language model. Subsequently, variants annotated as benign or likely benign in clinical databases were excluded from further analysis. In addition, variants corresponding to deletion or duplication events were removed to ensure that only single‐nucleotide substitutions suitable for downstream structural interpretation were retained. Following this filtering step, the retained coding substitutions were mapped to their corresponding reference SNP (rs) identifiers and evaluated at the amino acid level. This refinement process resulted in a curated set of LMNA missense variants with predicted functional relevance, which were subsequently subjected to structural, stability, and molecular interaction analyses.

**Table 1:** Prioritized loss‐of‐function (LOF) variants identified in the LMNA gene based on Evo2 delta scores and clinical annotations. Evo2 delta scores indicate evolutionary constraint, with more negative values suggesting higher functional impact. Clinical significance annotations were obtained from ClinVar, OMIM, UniProt, and gnomAD.

Variant_id	Gene	Evo2delta_score	Clinical_significance
156130730_C>GCTGGA	LMNA	−0.584676838	Likely pathogenic
156135936_G>GG	LMNA	−0.569397273	Pathogenic
156136286_G>GG	LMNA	−0.569293723	Pathogenic
156134500_T>A	LMNA	−0.01407316	Likely pathogenic; uncertain significance
156130658_G>A	LMNA	−0.01284181	Likely pathogenic; uncertain significance; pathogenic; not provided
156135968_G>T	LMNA	−0.01164725	Uncertain significance; conflicting interpretations of pathogenicity
156137642_T>G	LMNA	−0.001110738	Pathogenic; not provided; likely benign
156136054_G>A	LMNA	−0.001091894	Pathogenic; uncertain significance
156115017_G>C	LMNA	−0.001054347	Likely pathogenic; uncertain significance; pathogenic
156130688_C>G	LMNA	−0.001042782	Pathogenic; not provided
156115162_G>T	LMNA	−0.001036042	Likely pathogenic; uncertain significance; pathogenic
156115046_C>T	LMNA	−0.001035177	Pathogenic
156130669_C>G	LMNA	−0.00102031	Likely benign; uncertain significance; likely pathogenic
156136934_G>A	LMNA	−0.000993508	Likely pathogenic; pathogenic; not provided
156115162_G>C	LMNA	−0.000970455	Likely pathogenic; uncertain significance; pathogenic
156135902_T>G	LMNA	−0.000926193	Uncertain significance; conflicting interpretations of pathogenicity
156115066_C>A	LMNA	−0.00090373	Uncertain significance; pathogenic; not provided
156136915_C>T	LMNA	−0.000901995	Uncertain significance; likely benign; conflicting interpretations of pathogenicity
156115017_G>T	LMNA	−0.000881147	Likely pathogenic; uncertain significance; pathogenic
156115046_C>A	LMNA	−0.000866438	Pathogenic
156115110_C>T	LMNA	−0.00086384	Likely benign; conflicting interpretations of pathogenicity
156130624_A>C	LMNA	−0.000859849	Uncertain significance; pathogenic
156114991_C>T	LMNA	−0.000852875	Pathogenic likely pathogenic; likely pathogenic; pathogenic; not provided
156136286_G>‐	LMNA	−0.000825854	Pathogenic
156115094_T>A	LMNA	−0.000818202	Uncertain significance; pathogenic; not provided
156115001_G>A	LMNA	−0.000815442	Pathogenic; likely pathogenic
156115066_C>G	LMNA	−0.000759715	Uncertain significance; pathogenic; not provided
156115087_G>C	LMNA	−0.000746164	Pathogenic; not provided
156135902_T>C	LMNA	−0.000740563	Uncertain significance; conflicting interpretations of pathogenicity
156134949_G>T	LMNA	−0.000740436	Likely pathogenic; pathogenic
156130658_G>T	LMNA	−0.000717107	Likely pathogenic; uncertain significance; pathogenic; not provided
156115087_G>A	LMNA	−0.000682314	Pathogenic; not provided
156115112_A>G	LMNA	−0.000680648	Pathogenic; likely pathogenic
156134500_T>G	LMNA	−0.000667801	Likely pathogenic; uncertain significance
156115087_G>T	LMNA	−0.000665574	Pathogenic; not provided
156134490_A>T	LMNA	−0.000648853	Pathogenic
156134899_T>C	LMNA	−0.000642094	Uncertain significance; likely pathogenic
156136036_G>T	LMNA	−0.000641983	Pathogenic; not provided
156138593_G>A	LMNA	−0.000627712	Uncertain significance; conflicting interpretations of pathogenicity; likely benign
156135273_C>T	LMNA	−0.000625109	Uncertain significance; likely benign; conflicting interpretations of pathogenicity
156136423_A>C	LMNA	−0.000582352	Likely pathogenic; uncertain significance; not provided
156115105_A>G	LMNA	−0.000578241	Uncertain significance; likely pathogenic
156135903_G>‐	LMNA	−0.000576753	Pathogenic
156136422_A>G	LMNA	−0.000560977	Pathogenic; likely pathogenic; uncertain significance
156136240_C>A	LMNA	−0.000560182	Pathogenic; uncertain significance
156135913_G>A	LMNA	−0.000554133	Likely pathogenic; pathogenic; pathogenic likely pathogenic
156115163_A>G	LMNA	−0.00054946	Likely pathogenic
156135991_C>A	LMNA	−0.000536257	Likely benign; conflicting interpretations of pathogenicity; uncertain significance
156134500_T>‐	LMNA	−0.000528427	Pathogenic
156134458_G>A	LMNA	−0.000515329	Pathogenic; likely pathogenic; pathogenic likely pathogenic

Among the 50 prioritized variants in Table 1, clinical significance annotations were predominantly indicative of pathogenicity: 34 variants (68.0%) were annotated as pathogenic, 8 (16.0%) as likely pathogenic, and 8 (16.0%) had conflicting interpretations of pathogenicity in ClinVar; none of the 50 variants was annotated solely as benign, likely benign, or uncertain significance without an accompanying pathogenic or likely pathogenic annotation. This distribution may indicate that the Evo2‐based prioritization strategy substantially enriched for variants with clinical evidence of pathogenicity.

Among the Evo2‐prioritized variants carrying pathogenic or likely pathogenic ClinVar annotations, the most frequently represented condition was Charcot–Marie–Tooth disease Type 2, followed by cardiovascular/dilated cardiomyopathy phenotypes, Emery–Dreifuss muscular dystrophy, familial partial lipodystrophy (Dunnigan type), congenital muscular dystrophy, and Hutchinson–Gilford progeria syndrome (Supporting Information 2: Table S2). Notably, Charcot–Marie–Tooth Type 2 was overrepresented, whereas classic laminopathies such as Hutchinson–Gilford progeria and Emery–Dreifuss muscular dystrophy were comparatively underrepresented among the top Evo2‐prioritized variants.

### 3.2. Intrinsic Disorder Profile of the LMNA Protein

**Figure 1:**
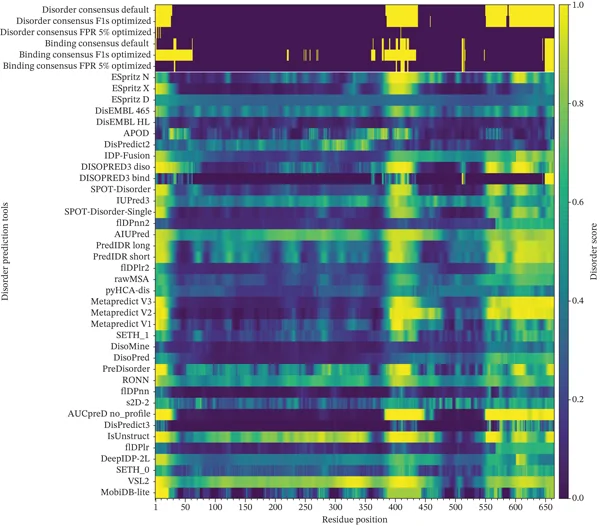
Heatmap of disorder prediction scores across the LMNA protein sequence. Each row represents a different prediction algorithm, and each column corresponds to an amino acid position. Color intensity indicates the predicted disorder score from 0 (ordered, dark blue) to 1 (disordered, yellow). Consensus predictors are shown at the top of the heatmap.

Figure 1 shows the residue‐level disorder scores across the LMNA protein sequence as predicted by multiple disorder predictors. The heatmap reveals clear heterogeneity in disorder propensity along the sequence. Elevated disorder is consistently observed in several regions, particularly toward the N‐terminal and C‐terminal segments, where multiple predictors converge on higher scores (Figure 1). In contrast, the central portion of the protein is predicted to be more ordered across most methods. Whereas individual predictors exhibit some variability in their residue‐level assignments, the consensus outputs demonstrate consistent patterns across the sequence, with extended structured domains interspersed with flexible regions.

### 3.3. Functional and Stability Prediction Results of LMNA Variants

The functional and stability impacts of 34 LMNA missense substitutions were evaluated using multiple in silico prediction tools, and the complete results are summarized in Supporting Information 3: Table S3. Overall, the majority of analyzed variants were consistently predicted to be functionally damaging, disease‐associated, and destabilizing across independent classifiers. PANTHER returned a prediction for 25 of the 34 variants, and all 25 scored variants were classified as damaging (22 [64.7%] “probably damaging” and 3 [8.8%] “possibly damaging”), whereas 9 (26.5%) could not be scored. PhD‐SNP classified 25 variants (73.5%) as “disease” and 9 (26.5%) as “neutral,” whereas E‐SNPs&GO classified 28 (82.4%) as “pathogenic” and only 6 (17.6%) as “benign.” Stability predictions showed a consistent tendency toward destabilization. INPS‐MD predicted 24 of 34 variants (70.6%) to be destabilizing or weakly destabilizing (11 destabilizing and 13 weakly destabilizing) and 10 (29.4%) to be neutral. DynaMut yielded a negative *Δ* *Δ* *G*, indicative of destabilization, for 22 variants (64.7%), with the remaining 12 (35.3%) showing mildly positive values. I‐Mutant2.0 predicted decreased stability for 28 variants (82.4%), and MUpro predicted decreased stability for 31 of 34 variants (91.2%). Pathogenicity assessment with MutPred2 further supported these findings: 24 variants (70.6%) exceeded the pathogenicity threshold (≥ 0.5), and 9 variants (26.5%)—L204Q, A57P, L204R, L245P, L59Q, R50G, A43E, N456D, and N456T—displayed particularly high scores above 0.8 (Table 2). Notably, the three variants selected for downstream structural and dynamic analyses (N456D, N456T, and G465D) were consistently classified as “probably damaging,” “disease‐associated,” and “pathogenic” (MutPred2 = 0.812, 0.810, and 0.694, respectively) and were predominantly predicted to reduce protein stability, while residing in structured regions suitable for reliable modeling and simulation. As a positive control, the known pathogenic globular domain variant M540T was assessed with the same panel of tools; whereas not all functional predictors flagged it (PhD‐SNP: neutral; no MutPred result), the stability predictors consistently indicated destabilization (INPS‐MD: destabilizing; DynaMut *Δ* *Δ* *G* = −0.603 kcal/mol, comparable with G465D at −0.604 kcal/mol; I‐Mutant2.0 and MUpro: decreased stability).

**Table 2:** Predicted disease association and protein stability effects of loss‐of‐function LMNA variants assessed by multiple bioinformatic tools.

SNP	Mutation	PANTHER	PhD‐SNP	E‐SNPs&GO	INPS‐MD	DynaMut	I‐Mutant2.0	MuPro	MutPred
rs60864230	R133L	Probably damaging	Disease	Pathogenic	Neutral	−0.081 kcal/mol	Decrease	Decrease stability	0.597
rs754097769	T157M	Possibly damaging	Neutral	Benign	Neutral	−0.121 kcal/mol	Decrease	Decrease stability	0.151
rs2102817667	A43E	Probably damaging	Disease	Pathogenic	Weakly destabilizing	0.237 kcal/mol	Decrease	Decrease stability	0.949
rs2102886618	L313P	Probably damaging	Disease	Pathogenic	Destabilizing	−0.46 kcal/mol	Decrease	Decrease stability	0.731
rs1553265180	L204Q	—	Disease	Pathogenic	Destabilizing	−0.251 kcal/mol	Decrease	Decrease stability	0.855
rs267607571	R190Q	Probably damaging	Disease	Pathogenic	Destabilizing	−0.217 kcal/mol	Decrease	Decrease stability	0.743
rs56816490	E317K	Probably damaging	Disease	Pathogenic	Weakly destabilizing	0.28 kcal/mol	Decrease	Decrease stability	0.571
rs267607599	N456D	Probably damaging	Disease	Pathogenic	Neutral	0.031 kcal/mol	Decrease	Decrease stability	0.812
rs60992550	N456T	Probably damaging	Disease	Pathogenic	Neutral	0.408 kcal/mol	Increase	Decrease stability	0.810
rs61282106	G465D	Probably damaging	Disease	Pathogenic	Weakly destabilizing	−0.604 kcal/mol	Decrease	Decrease stability	0.694
rs267607547^a^	M540T^a^	Possibly damaging	Neutral	Pathogenic	Destabilizing	−0.603 kcal/mol	Decrease	Decrease stability	—

*Note:* “—” indicates that no prediction result could be obtained for this variant using the corresponding tool.

^a^M540T (rs267607547) is included as a positive control, a known pathogenic variant localized within the globular Ig‐fold domain, rather than an Evo2‐prioritized candidate.

### 3.4. Physicochemical Characterization of LMNA Variants

A detailed analysis of physicochemical parameters for the LMNA protein and its variants was conducted using the ExPASy ProtParam tool, and the results are provided in Supporting Information 4: Table S4. All variants, including the wild type, were predicted to be unstable according to the II, with scores exceeding the standard threshold of 40. The II ranged from 51.14 (R335L) to 52.87 (T157R), indicating a consistent classification of the LMNA protein and its variants as unstable proteins (Table 3, Supporting Information 4: Table S4). In terms of molecular weight, only minor fluctuations were observed across variants, with the wild‐type LMNA at 74,139.49 Da, and most substitutions causing negligible deviations. Similarly, theoretical pIs exhibited minor shifts, ranging from 6.44 to 6.94, with E317K displaying the highest pI among all variants. The total number of charged residues (sum of Asp + Glu and Arg + Lys) remained relatively constant, typically between 106–108 negative and 103–105 positive residues. The aliphatic index showed minor variations across the variants, ranging from 74.11 (L204Q and L313P) to 75.29 (R133L and R335L), indicating relatively constant hydrophobic content. All variants exhibited negative GRAVY scores, consistent with the hydrophilic nature of LMNA and its mutant forms. GRAVY values ranged from −0.850 to −0.875, with L204R being the most hydrophilic (Table 2, Supporting Information 4: Table S4).

**Table 3:** Comparative analysis of molecular weight, isoelectric point (pI), charge distribution, instability index, aliphatic index, and GRAVY values for the wild‐type LMNA protein and its variants.

Variant	Molecular weight	Theoretical pI	Total number of negatively charged residues (Asp + Glu)	Total number of positively charged residues (Arg + Lys)	The instability index (II)	Aliphatic index	GRAVY
Normal	74,139.49	6.57	107	104	52.00/unstable	74.70	−0.863
R133L	74,096.46	6.44	107	103	52.00/unstable	75.29	−0.850
T157M	74,169.58	6.57	107	104	52.00/unstable	74.70	−0.859
A43E	74,197.53	6.44	108	104	52.00/unstable	74.55	−0.871
L313P	74,123.45	6.57	107	104	52.58/unstable	74.11	−0.871
L204Q	74,154.46	6.57	107	104	52.58/unstable	74.11	−0.874
R190Q	74,111.43	6.44	107	103	51.32/unstable	74.70	−0.861
E317K	74,138.55	6.94	106	105	52.00/unstable	74.70	−0.864
N456D	74,140.47	6.44	108	104	51.33/unstable	74.70	−0.863
N456T	74,126.49	6.57	107	104	51.46/unstable	74.70	−0.859
G465D	74,183.50	6.44	108	104	52.13/unstable	74.55	−0.867

Notably, the prioritized variants N456D, N456T, and G465D all shared physicochemical profiles largely comparable to the wild‐type protein. For instance, all three exhibited an II above 51. N456D had a slightly increased number of negatively charged residues (108) compared with the wild type (107), whereas G465D showed the highest molecular weight (74,183.50 Da) among all variants (Table 3, Supporting Information 4: Table S4). These observations indicate that the potential pathogenic effects of these substitutions are unlikely to stem from substantial alterations in global protein properties, but rather from local structural perturbations that may affect protein conformation.

### 3.5. Structural Impact and Conservation Analysis of LMNA Variants

Structural and conservation‐based analyses of LMNA variants were performed using the HOPE server, and the findings are summarized in Supporting Information 5: Table S5. The assessment involved evaluating amino acid substitutions in terms of size, charge, hydrophobicity changes, potential structural disruptions, and conservation‐based pathogenicity predictions. Out of the analyzed mutations, a substantial portion, approximately half of the variants, were predicted to be damaging to the protein structure or function, often due to loss of H‐bonds, alteration of charge, or disruption of hydrophobic interactions. Specifically, variants such as T157M, T157R, L204Q, R190Q, E317K, A57P, L313P, A43E, R335L, A57S, D364N, S143F, and G465D were all classified as “probably yes” under the “damaging to the protein conservation” column (Table 4, Supporting Information 5: Table S5). Among the three key variants studied across the full pipeline, N456D, N456T, and G465D were all also predicted as damaging. Both N456D and G465D were described as potentially leading to protein folding problems, whereas N456T was associated with loss of hydrogen bonding and disturbed folding. These outputs reinforce the likelihood that these substitutions affect the native conformation and stability of the LMNA protein. In contrast, several variants, including R133L, T157K, E82G, E65G, R50G, L245P, and K122Q, were classified as “probably no,” suggesting minimal impact on structural conservation or folding (Table 4, Supporting Information 5: Table S5).

**Table 4:** Comparative analysis of physicochemical changes and predicted structural effects induced by selected LMNA missense variants based on in silico evaluation.

Variant	Wild‐type amino acid			Mutant‐type amino acid		
	Size	Charge	Hydrophobicity	Charge	Mutant residue	Damaging to protein conservation
R133L	>	Positive	<	Neutral	Loss of hydrogen bonds, disturb structure	Probably no
T157M	<	—	<	—	Loss of hydrogen bonds, disturb structure	Probably yes
A43E	<	Neutral	>	Negative	Loss of hydrophobic interactions	Probably yes
L313P	>	—	—	—	This might lead to loss of interactions	Probably yes
L204Q	<	—	>	—	Disturb interactions	Probably yes
R190Q	>	Positive	—	Neutral	Loss of external interactions, disturb the ionic interaction	Probably yes
E317K	<	Negative	—	Positive	Might lead to bumps	Probably yes
N456D	—	Neutral	—	Negative	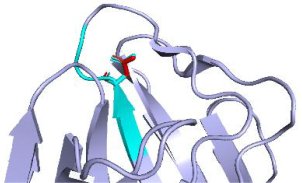	Probably yes
					Can lead to protein folding problems	
N456T	>	—	<	—	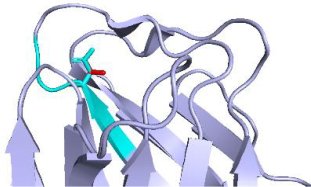	Probably yes
					Loss of hydrogen bonds, disturb correct folding	
G465D	<	Neutral	>	Negative	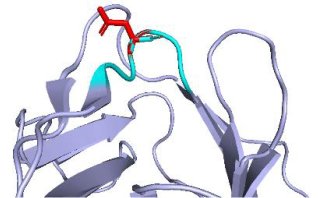	Probably yes
					Lead to protein folding problems	

*Note:* “—” indicates that no result could be obtained for this parameter using the corresponding computational analysis. Turquoise indicates the wild‐type residue, whereas red indicates the mutant residue in structural representations.

### 3.6. Molecular Docking Analysis of Wild‐Type and Mutant LMNA–Lonafarnib Complexes

Molecular docking analysis was performed to compare the binding mode of lonafarnib with the wild‐type LMNA protein and the N456D, N456T, G465D, and M540T variants (Table 5). The predicted binding affinities were within a narrow range across all systems: −7.071 kcal/mol (blind) and −7.705 kcal/mol (flexible) for N456D, −6.991 kcal/mol (both docking modes) for N456T, −7.394 kcal/mol (blind) and −7.779 kcal/mol (flexible) for G465D, and −7.02 kcal/mol (blind) and −7.123 kcal/mol (flexible) for M540T. The wild‐type complex exhibited a diverse interaction profile consisting of conventional and carbon–hydrogen bonds together with *π*–*π* T‐shaped, alkyl, *π*‐alkyl, and van der Waals interactions. The major interacting residues included GLN517, LEU478, HIS506, TYR481, TRP514, PRO484, ALA516, PRO485, LEU512, LYS515, ARG482, PHE483, THR480, LEU479, and VAL513 (Table 5). The N456D mutant largely preserved the interaction pattern observed in the wild‐type complex. Conventional H‐bonds with GLN517, THR480, and LEU478, together with carbon–hydrogen bonding involving HIS506, indicated that the overall binding orientation remained highly conserved. Similarly, the N456T variant retained the wild‐type interaction pattern, forming conventional H‐bonds with GLN517, THR480, and LEU478 and preserving the aromatic *π*–*π* (TYR481) and *π*‐alkyl (TRP514, PRO485, and LEU512) contacts characteristic of the wild‐type and N456D complexes (Table 5).

**Table 5:** Summary of interaction profiles for wild‐type, N456T, N456D, G465D, and M540T docking complexes.

Interaction type	Wild type	N456T	N456D	G465D	M540T
Conventional hydrogen bond	GLN517, LEU478	GLN517, THR480, LEU478	GLN517, THR480, LEU478	ASN532, TRP467	VAL513
Carbon–hydrogen bond	HIS506	HIS506	HIS506	—	—
*π*‐Cation	—	—	—	GLU536, ARG439	—
*π*–*π* T‐shaped	TYR481	TYR481	TYR481	—	—
Alkyl	PRO484, ALA516	PRO484, ALA516	PRO484, ALA516	VAL538	PRO484, ALA516
*π*‐Alkyl	TRP514, PRO485, LEU512	TRP514, PRO485, LEU512	TRP514, PRO485, LEU512	—	—
van der Waals	LYS515, VAL513, ARG482, PHE483, THR480, LEU479	LYS515, VAL513, ARG482, PHE483, LEU479	LYS515, VAL513, ARG482, PHE483, HIS506, LEU479	GLN462, ASN466, ASP465, SER463, SER533, MET464, SER437, GLY438	HIS506, TYR481, TRP514, LEU512, LYS515

In contrast, the G465D mutation produced a substantially altered interaction profile. The loss of aromatic *π*–*π* and *π*‐alkyl interactions was accompanied by the emergence of *π*‐cation interactions involving GLU536 and ARG439, together with an alkyl interaction with VAL538. Moreover, the ligand established H‐bonds with ASN532 and TRP467 and formed multiple van der Waals contacts with GLN462, ASN466, ASP465, SER463, SER533, MET464, SER437, and GLY438. The M540T mutant displayed the simplest interaction profile among all complexes. A single conventional H‐bond with VAL513 was retained, and the remaining contacts comprised alkyl interactions with PRO484 and ALA516, together with van der Waals interactions involving HIS506, TYR481, TRP514, LEU512, and LYS515.

### 3.7. MD Simulation Results

**Figure 2:**
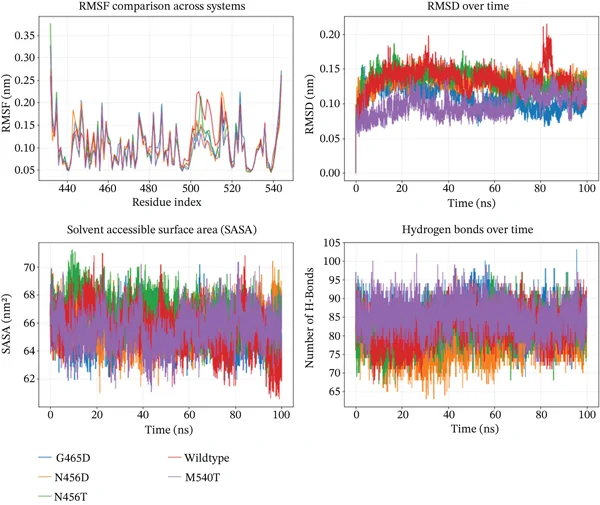
Comparative molecular dynamics analysis of the wild‐type LMNA–lonafarnib complex, the known pathogenic variant M540T, and the predicted variants N456T, N456D, and G465D over a 100 ns simulation. Four structural and interaction‐based MD metrics are shown: root mean square deviation (RMSD), root mean square fluctuation (RMSF), solvent accessible surface area (SASA), and the number of protein–ligand hydrogen bonds.

Figure 2 summarizes the MD simulation outputs over 100 ns for the wild‐type LMNA–lonafarnib complex and four mutant systems (N456T, N456D, G465D, and M540T). The RMSD plots show that all complexes reached structural stability after the initial equilibration phase, with only minor fluctuations observed throughout the simulations. Among the analyzed systems, the G465D and M540T variants exhibited relatively lower average RMSD values, indicating stable conformational behavior during the production phase. RMSF analysis revealed generally similar residue‐level flexibility profiles across all systems, although localized differences were observed in several residue regions, particularly for the N456D, G465D, and M540T variants. The SASA remained relatively stable over the 100 ns simulations, suggesting that none of the mutations caused substantial changes in the overall compactness of the protein. The number of H‐bonds fluctuated throughout the simulations but remained within a comparable range among all systems.

**Figure 3:**
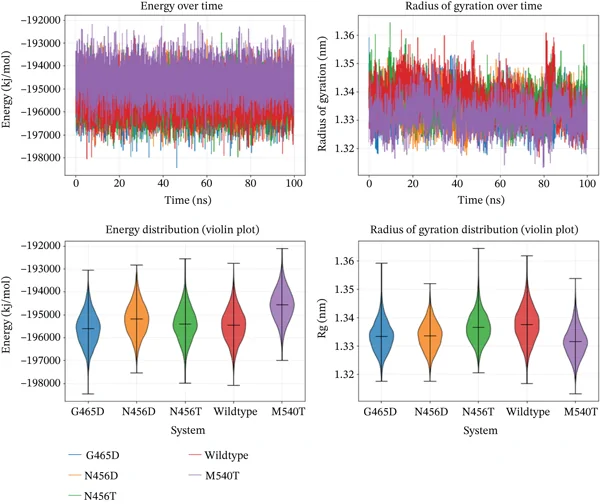
Potential energy and radius of gyration analysis of the wild‐type LMNA–lonafarnib complex, the known pathogenic variant M540T, and the predicted variants N456T, N456D, and G465D.

Figure 3 presents the total potential energy and structural compactness, represented by the Rg, of the wild‐type LMNA–lonafarnib complex and four mutant systems (N456T, N456D, G465D, and M540T) throughout the 100 ns MD simulations. The potential energy trajectories remained stable for all systems, indicating that each complex reached and maintained an energetically equilibrated state during the simulation. The energy distribution violin plots further demonstrated comparable energy profiles among the complexes. Although slight differences in the mean energy values were observed, the M540T complex exhibited a marginally less negative potential energy distribution than the other systems, whereas G465D, N456T, N456D, and the wild type displayed largely overlapping energy ranges. The Rg remained stable throughout the simulations for all complexes, indicating preservation of the overall structural compactness without evidence of significant unfolding or collapse. The Rg distribution plots showed only minor differences among the systems, with the M540T complex exhibiting a slightly lower average Rg value, suggesting a modest increase in structural compactness relative to the other variants.

#### 3.7.1. PCA and FES Mapping

**Figure 4:**
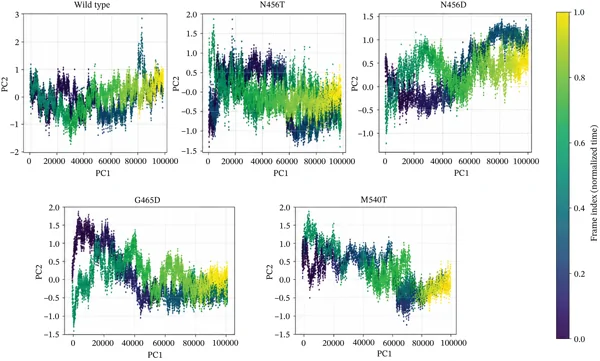
Principal component analysis (PCA) of LMNA–lonafarnib complexes over time. The plots reveal the conformational evolution and dynamic sampling behavior of wild‐type and mutant complexes.

**Figure 5:**
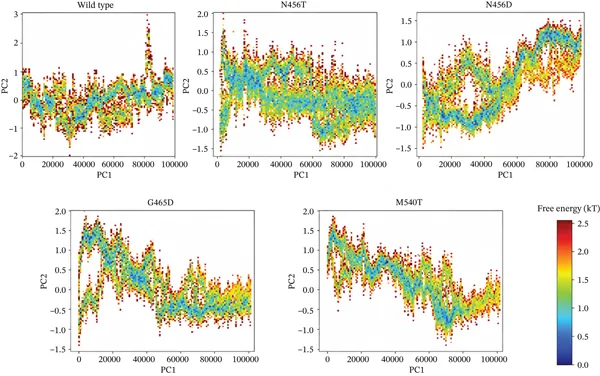
Free‐energy surface (FES) maps based on principal component analysis (PCA) of wild‐type and mutant LMNA–lonafarnib complexes. Each map shows the conformational space sampled during the 100 ns simulation, projected onto PC1 and PC2. The color scale indicates relative free‐energy levels in kT, with darker colors (blue) representing lower energy, more stable regions, and warmer colors (red) indicating higher energy, less favorable conformations.

Figures 4 and 5 summarize the PCA‐based conformational sampling (Figure 4) and the corresponding free‐energy landscapes (Figure 5) of the wild‐type LMNA–lonafarnib complex and four mutant systems (N456T, N456D, G465D, and M540T), projected onto the first two principal components (PC1 and PC2) over 100 ns of MD simulation. The wild‐type complex exhibited a relatively confined conformational space, characterized by a gradual and continuous transition along PC1 over time (Figure 4). The N456T variant displayed a broader distribution across both PC1 and PC2, indicating increased flexibility and more extensive exploration of the conformational landscape. The N456D mutant exhibited a bifurcated trajectory, with early and late simulation frames occupying distinct regions of the principal component space, consistent with a transition between two dominant conformational states. The G465D variant also sampled a broad conformational space but followed a more continuous transition toward lower PC2 values as the simulation progressed. In contrast, the M540T mutant demonstrated a progressive shift from positive to negative PC2 values while maintaining a relatively continuous trajectory along PC1. Compared with G465D and N456T, the M540T system occupied a more localized conformational region during the later stages of the simulation, indicating moderately restricted conformational sampling.

The corresponding FES maps further supported these observations (Figure 5). The wild‐type complex exhibited a relatively narrow and compact free‐energy basin centered primarily along PC1 with limited dispersion along PC2, reflecting a stable conformational ensemble dominated by a single major energy minimum. The N456T mutant displayed a broader and more fragmented energy landscape containing several shallow local minima, indicative of increased conformational flexibility and sampling of multiple metastable states. The N456D variant presented two well‐defined low‐energy basins separated along the PC1 axis, suggesting the coexistence of two dominant conformational populations connected by a transition during the simulation. The G465D mutant exhibited the most dispersed free‐energy landscape, characterized by multiple spatially separated minima distributed across both principal components, reflecting enhanced conformational heterogeneity and the absence of a single dominant structural state. The M540T mutant displayed an intermediate free‐energy profile, with a principal low‐energy basin accompanied by several smaller neighboring minima. Compared with G465D, its energy landscape was less fragmented, whereas it remained broader than that of the wild‐type complex.

## 4. Discussion

This study presents an integrated in silico framework to elucidate how computationally prioritized missense variants in the LMNA gene affect protein structure, stability, and dynamics. By combining evolutionary analysis, disorder prediction, physicochemical profiling, structural modeling, molecular docking, MD simulations, and free‐energy landscape analysis, we provide a multiscale view of how sequence‐level alterations propagate into structural and functional consequences at the protein level.

The initial prioritization of LMNA variants was guided by evolutionary conservation and functional relevance rather than solely by clinical annotation (Table 1). This strategy is supported by accumulating evidence that pathogenic LMNA mutations are predominantly localized within protein‐coding regions and exert their effects through perturbation of Lamin A structure, assembly, and mechanical stability rather than transcriptional dysregulation alone [1, 3, 6]. Mapping these Evo2‐prioritized variants to their ClinVar disease annotations further showed that the prioritized set was enriched for peripheral nerve and cardiac/skeletal muscle laminopathies—most notably Charcot–Marie–Tooth disease Type 2—whereas classic progeroid and Emery–Dreifuss phenotypes were comparatively underrepresented (Supporting Information 2: Table S2), indicating that evolutionary constraint–based scoring preferentially captures a specific subset of the laminopathy spectrum. Consistent with this, disorder profiling revealed a heterogeneous distribution of disorder across the LMNA sequence, with increased disorder propensity at the N‐ and C‐terminal regions (Figure 1). These regions are known to contribute to nuclear envelope plasticity and protein–protein interactions but are inherently unsuitable for structure‐based modeling due to their conformational flexibility [38, 39, 51]. Consequently, variants located within such intrinsically disordered segments were excluded from downstream analyses to ensure robust structural interpretation.

Although several variants exhibited comparable pathogenicity and stability prediction scores, only N456D, N456T, and G465D were selected for detailed analysis based on their localization within ordered regions amenable to structural modeling. This decision aligns with previous studies showing that mutations within structured lamin domains are more likely to disrupt filament assembly, nuclear mechanics, and chromatin tethering [6, 7, 52]. Direct experimental support for this mechanism comes from studies of other Ig‐fold domain variants. The EDMD‐associated mutation R453W was shown to mechanically destabilize the Ig‐fold domain using single‐molecule force spectroscopy and steered MD [53]. The muscular dystrophy–associated mutation W514R was shown to alter the structure and dynamics of the same domain using MD simulation [54]. Physicochemical analysis revealed subtle but consistent alterations in charge distribution, hydrophobicity, and stability indices (Tables 3 and 2). Such moderate physicochemical shifts are characteristic of laminopathies, where cumulative destabilization rather than drastic unfolding leads to progressive cellular dysfunction. Structural impact analysis indicated that the selected mutations perturb local residue packing and hydrogen bonding networks (Table 4), consistent with previous reports linking LMNA mutations to impaired filament assembly and nuclear envelope integrity [8, 16, 55]. Notably, these disruptions were observed across multiple independent prediction tools, reinforcing the robustness of the findings and minimizing tool‐specific bias. The convergence of predictions suggests that even subtle amino acid substitutions within structured regions can destabilize Lamin A architecture and compromise its mechanical role.

The functional relevance of these structural alterations was further explored through molecular docking analyses using lonafarnib, a clinically approved farnesyltransferase inhibitor employed in Hutchinson–Gilford progeria syndrome [5], used here as a reference ligand. Whereas all variants retained the ability to bind lonafarnib, subtle differences in interaction geometry and residue involvement were observed between wild‐type and mutant complexes (Table 5). Furthermore, inclusion of the clinically established M540T variant as a positive control provided an additional reference for interpreting the computational findings. Although M540T retained a simpler interaction network than the wild‐type complex, its overall docking behavior remained comparable in terms of binding affinity, supporting the notion that mutation‐associated changes are reflected primarily in the local interaction network rather than in large differences in docking scores. This observation also supports the suitability of comparing the newly prioritized variants against a clinically recognized globular domain mutation. A critical point to consider is that lonafarnib is not a direct ligand of Lamin A, but rather a farnesyltransferase inhibitor used clinically to modulate Prelamin A processing [41]. Accordingly, the docking and MD results presented here should not be interpreted as evidence of a direct pharmacological interaction between lonafarnib and LMNA. Instead, the ligand was employed as a reference molecular probe to investigate how mutation‐induced structural perturbations reshape the local interaction landscape within the globular domain of Lamin A. This strategy allows for a controlled comparison between wild‐type and mutant systems under identical simulation conditions, thereby enabling the identification of mutation‐dependent differences in binding geometry, interaction networks, and conformational stability. This approach is particularly valuable in the context of laminopathies, where disease mechanisms are primarily driven by structural instability and aberrant protein processing rather than classical ligand‐binding dysfunction. Therefore, the observed differences in interaction patterns should be interpreted as indicators of altered structural environments rather than as direct measures of drug efficacy. In particular, the G465D variant displayed altered binding orientations and interaction networks, suggesting that mutation‐induced local rearrangements may alter the local interaction landscape within the protein structure. For G465D specifically, lonafarnib was displaced from the canonical wild‐type binding pocket and engaged an alternative set of contact residues, forming new H‐bond and *π*‐cation interactions with ASN532, TRP467, GLU536, and ARG439 while losing the aromatic *π*–*π* and *π*‐alkyl contacts that stabilized the wild‐type, N456D, and N456T complexes (Table 5). In contrast, the N456D and N456T variants largely retained the wild‐type interaction network, indicating that the G465D substitution induces a more extensive local remodeling of the ligand‐binding environment. These observations are consistent with previous reports indicating that LMNA mutations can affect drug–protein interactions by reshaping the local structural environment rather than abolishing binding entirely [17]. The MD simulations further demonstrated that all LMNA–lonafarnib complexes remained structurally stable throughout the 100 ns simulation period. Although mutation‐dependent differences were observed in residue flexibility and H‐bond occupancy, RMSD, RMSF, SASA, potential energy, and Rg collectively indicated that none of the investigated substitutions induced large‐scale structural destabilization. Notably, the pathogenic control variant M540T exhibited stability comparable to that of the newly prioritized variants, supporting the interpretation that pathogenic LMNA mutations primarily perturb local structural dynamics rather than causing global unfolding of the globular domain. PCA and free‐energy landscape analysis further supported these observations by revealing mutation‐dependent differences in conformational sampling. Whereas the wild‐type complex occupied a relatively compact conformational basin, the mutant systems explored broader conformational spaces characterized by multiple metastable states (Figures 4 and 5). Among them, G465D exhibited the greatest conformational heterogeneity, whereas M540T maintained a comparatively more restricted conformational distribution despite its pathogenic status. These findings may suggest that disease‐associated LMNA substitutions primarily remodel local conformational dynamics and interaction networks while preserving the protein′s overall structural integrity. Complementary analyses of SASA and hydrogen bonding patterns further supported the presence of altered compactness and weakened stabilizing interactions. Importantly, PCA and FES mapping provided a complementary view of these effects, revealing that mutant systems occupied broader structural rearrangements and more fragmented energy landscapes than the wild‐type complex (Figures 4 and 5). The presence of multiple low‐energy basins reflects enhanced conformational heterogeneity, a feature commonly associated with pathogenic variants and impaired structural robustness [49, 50, 56]. Collectively, these findings support a model in which specific *LMNA* variants induce localized structural perturbations that propagate into altered dynamic behavior, potentially compromising the mechanical and binding properties of Lamin A.

## 5. Conclusion

In this study, an integrated computational approach was applied to investigate the structural and dynamic consequences of LMNA variants with potential pathogenic relevance. By combining evolutionary analysis, structural modeling, molecular docking, MD simulations, and free‐energy landscape evaluation, rather than inducing global structural instability, the investigated mutations primarily altered local interaction networks, conformational sampling, and dynamic behavior, with G465D exhibiting the most pronounced reorganization of ligand interactions and conformational landscape. In particular, mutations located within structurally defined globular regions, such as N456D, N456T, and G465D, were associated with measurable changes in stability, conformational dynamics, and ligand interaction patterns. These findings emphasize the importance of incorporating structural information, rather than pathogenicity scores alone, when prioritizing LMNA variants. At the same time, the study is limited by its computational nature and reliance on a single reference ligand, highlighting the need for future experimental validation. Overall, this work provides a systematic framework to link sequence variation with structural and dynamic effects in Lamin A, offering mechanistic hypotheses for future studies aimed at clarifying the molecular basis of laminopathies.

## Funding

No funding was received for this manuscript.

## Ethics Statement

The study does not include human or animal experiments.

## Consent

Informed consent was obtained from all individual participants included in the study.

## Conflicts of Interest

The authors declare no conflicts of interest.

## Supporting Information

Additional supporting information can be found online in the Supporting Information section.

## Supporting information


**Supporting Information 1** Table S1: ClinVar‐annotated pathogenic LMNA variants considered positive controls, together with their domain localization (*α*‐helical rod domain vs. C‐terminal Ig‐fold domain). This table documents the rationale for selecting M540T as the positive control for the docking and molecular dynamics analyses.

**Supporting Information 2** Table S2: Disease phenotypes associated with the Evo2‐prioritized variants carrying pathogenic or likely pathogenic ClinVar annotations, showing the distribution of laminopathy subtypes across the prioritized variant set.

**Supporting Information 3** Table S3: Complete functional and stability prediction results for the 34 prioritized LMNA missense substitutions, obtained with SIFT, PANTHER‐PSEP, PhD‐SNP, E‐SNPs&GO, INPS‐MD, DynaMut, I‐Mutant2.0, MUpro, and MutPred2.

**Supporting Information 4** Table S4: Physicochemical parameters of the wild‐type and mutant LMNA proteins computed with the ExPASy ProtParam tool, including molecular weight, theoretical isoelectric point, number of charged residues, instability index, aliphatic index, and GRAVY value.

**Supporting Information 5** Table S5: Full output of the HOPE server for the analyzed LMNA variants, summarizing the predicted changes in residue size, charge, and hydrophobicity together with the predicted structural consequences and conservation‐based damaging assessments.

## Data Availability

All data generated or analyzed during this study are included in this article and its supporting information.
