# Supplementary material for: Computational Characterization of Pathogenic LMNA Missense Variants: Structural Instability, Altered Binding, and Conformational Dynamics
Source: Hum Mutat. 2026 Aug 26;2026:2444803. doi: 10.1155/humu/2444803 (PMC13508025; doi:10.1155/humu/2444803)
Supplement: Supplementary file 1 [file HUMU-2026-2444803-s001.docx]

**Supplementary Table S4.** Comparative analysis of molecular weight, isoelectric point (pI), charge distribution, instability index, aliphatic index, and GRAVY values for the wild-type LMNA protein and its variants.

| Variant | Molecular weight | Theoretical PI | Total number of negatively charged residues (Asp + Glu) | Total number of positively charged residues (Arg + Lys) | The instability index (II) | Aliphatic index | GRAVY |  |
| --- | --- | --- | --- | --- | --- | --- | --- | --- |
|  |  |  |  |  |  |  |  |  |
| Normal | 74139.49 | 6.57 | 107 | 104 | 52.00 / unstable | 74.70 | -0.863 |  |
| R133L | 74096.46 | 6.44 | 107 | 103 | 52.00 / unstable | 75.29 | -0.850 |  |
| T157M | 74169.58 | 6.57 | 107 | 104 | 52.00 / unstable | 74.70 | -0.859 |  |
| T157K | 74166.56 | 6.73 | 107 | 105 | 51.88 / unstable | 74.70 | -0.868 |  |
| T157R | 74194.57 | 6.73 | 107 | 105 | 52.87 / unstable | 74.70 | -0.869 |  |
| L204Q | 74154.46 | 6.57 | 107 | 104 | 52.58 / unstable | 74.11 | -0.874 |  |
| R190Q | 74111.43 | 6.44 | 107 | 103 | 51.32 / unstable | 74.70 | -0.861 |  |
| E82G | 74067.43 | 6.73 | 106 | 104 | 51.86 / unstable | 74.70 | -0.858 |  |
| E317K | 74138.55 | 6.94 | 106 | 105 | 52.00 / unstable | 74.70 | -0.864 |  |
| N456D | 74140.47 | 6.44 | 108 | 104 | 51.33 / unstable | 74.70 | -0.863 |  |
| N456T | 74126.49 | 6.57 | 107 | 104 | 51.46 / unstable | 74.70 | -0.859 |  |
| A57P | 74165.53 | 6.57 | 107 | 104 | 51.96 / unstable | 74.55 | -0.868 |  |
| E65G | 74067.43 | 6.73 | 106 | 104 | 51.30 / unstable | 74.70 | -0.858 |  |
| L204R | 74182.52 | 6.73 | 107 | 105 | 52.00 / unstable | 74.11 | -0.875 |  |
| L245P | 74123.45 | 6.57 | 107 | 104 | 52.09 / unstable | 74.11 | -0.871 |  |
| L59Q | 74154.46 | 6.57 | 107 | 104 | 51.71 / unstable | 74.11 | -0.874 |  |
| R28Q | 74111.43 | 6.44 | 107 | 103 | 51.89 / unstable | 74.70 | -0.861 |  |
| R50G | 74040.35 | 6.44 | 107 | 103 | 51.21 / unstable | 74.70 | -0.857 |  |
| L313P | 74123.45 | 6.57 | 107 | 104 | 52.58 / unstable | 74.11 | -0.871 |  |
| A43E | 74197.53 | 6.44 | 108 | 104 | 52.00 / unstable | 74.55 | -0.871 |  |
| R133Q | 74111.43 | 6.44 | 107 | 103 | 52.00 / unstable | 74.70 | -0.861 |  |
| R335L | 74096.46 | 6.44 | 107 | 103 | 51.14 / unstable | 75.29 | -0.850 |  |
| E82Q | 74138.50 | 6.73 | 106 | 104 | 52.12 / unstable | 74.70 | -0.863 |  |
| A57S | 74155.49 | 6.57 | 107 | 104 | 52.00 / unstable | 74.55 | -0.867 |  |
| G602S | 74169.52 | 6.57 | 107 | 104 | 52.58 / unstable | 74.70 | -0.864 |  |
| I63V | 74125.46 | 6.57 | 107 | 104 | 51.88 / unstable | 74.55 | -0.863 |  |
| R190Q | 74111.43 | 6.44 | 107 | 103 | 51.32 / unstable | 74.70 | -0.861 |  |
| D364N | 74138.50 | 6.73 | 106 | 104 | 52.67 / unstable | 74.70 | -0.863 |  |
| E33D | 74125.46 | 6.57 | 107 | 104 | 51.71 / unstable | 74.70 | -0.863 |  |
| S143F | 74199.59 | 6.57 | 107 | 104 | 51.55 / unstable | 74.70 | -0.858 |  |
| L137V | 74125.46 | 6.57 | 107 | 104 | 52.00 / unstable | 74.55 | -0.862 |  |
| G465D | 74183.50 | 6.44 | 108 | 104 | 52.13 / unstable | 74.55 | -0.867 |  |
| K122Q | 74139.45 | 6.44 | 107 | 103 | 51.89 / unstable | 74.70 | -0.862 |  |
| R25C | 74086.44 | 6.44 | 107 | 103 | 52.00 / unstable | 74.70 | -0.852 |  |
| I63L | 74139.49 | 6.57 | 107 | 104 | 52.00 / unstable | 74.70 | -0.864 |  |
