# Supplementary material for: Computational Characterization of Pathogenic LMNA Missense Variants: Structural Instability, Altered Binding, and Conformational Dynamics
Source: Hum Mutat. 2026 Aug 26;2026:2444803. doi: 10.1155/humu/2444803 (PMC13508025; doi:10.1155/humu/2444803)
Supplement: Supplementary file 2 [file HUMU-2026-2444803-s002.docx]

**Supplementary Table S3** Predicted disease association and protein stability effects of loss-of-function LMNA variants assessed by multiple bioinformatic tools.

| SNP | MUTASYON | Panther | PhD-SNP | E-SNPs&GO | INPS-MD | DynaMut | I-mutant2.0 | Mu-Pro | MutPred |
| --- | --- | --- | --- | --- | --- | --- | --- | --- | --- |
| rs754097769 | T157M | Possibly damaging | Neutral | Benign | Neutral | -0.121 kcal/mol | Decrease | Decrease stability | 0.151 |
| rs754097769 | T157K | Possibly damaging | Neutral | Benign | Neutral | 0.24 kcal/mol | Decrease | Decrease stability | 0.357 |
| rs754097769 | T157R | Possibly damaging | Neutral | Benign | Neutral | 0.153 kcal/mol | Decrease | Decrease stability | 0.379 |
| rs1553265180 | L204Q | a | Disease | Pathogenic | Destabilizing | -0.251 kcal/mol | Decrease | Decrease stability | 0.855 |
| rs267607571 | R190Q | Probably damaging | Disease | Pathogenic | Destabilizing | -0.217 kcal/mol | Decrease | Decrease stability | 0.743 |
| rs2527833277 | E82G | a | Disease | Pathogenic | Destabilizing | -1.022 kcal/mol | Decrease | Decrease stability | 0.785 |
| rs56816490 | E317K | Probably damaging | Disease | Pathogenic | Weakly destabilizing | 0.28 kcal/mol | Decrease | Decrease stability | 0.571 |
| rs267607599 | N456D | Probably damaging | Disease | Pathogenic | Neutral | 0.031 kcal/mol | Decrease | Decrease stability | 0.812 |
| rs60992550 | N456T | Probably damaging | Disease | Pathogenic | Neutral | 0.408 kcal/mol | Increase | Decrease stability | 0.810 |
| rs60864230 | R133L | Probably damaging | Disease | Pathogenic | Neutral | -0.081 kcal/mol | Decrease | Decrease stability | 0.597 |
| rs28928903 | A57P | Probably damaging | Disease | Pathogenic | Destabilizing | -0.234 kcal/mol | Increase | Decrease stability | 0.854 |
| rs2102818172 | E65G | Probably damaging | Neutral | Pathogenic | Weakly destabilizing | -0.986 kcal/mol | Decrease | Decrease stability | 0.609 |
| rs1553265180 | L204R | a | Disease | Pathogenic | Destabilizing | -0.153 kcal/mol | Decrease | Decrease stability | 0.909 |
| rs2527973813 | L245P | a | Disease | Pathogenic | Destabilizing | -0.783 kcal/mol | Decrease | Decrease stability | 0.917 |
| rs58922911 | L59Q | Probably damaging | Disease | Pathogenic | Destabilizing | -0.266 kcal/mol | Decrease | Decrease stability | 0.936 |
| rs886043109 | R28Q | a | Disease | Pathogenic | Destabilizing | 0.102 kcal/mol | Decrease | Decrease stability | 0.766 |
| rs59931416 | R50G | Probably damaging | Disease | Pathogenic | Destabilizing | -0.341 kcal/mol | Decrease | Decrease stability | 0.934 |
| rs2102886618 | L313P | Probably damaging | Disease | Pathogenic | Destabilizing | -0.46 kcal/mol | Decrease | Decrease stability | 0.731 |
| rs2102817667 | A43E | Probably damaging | Disease | Pathogenic | Weakly destabilizing | 0.237 kcal/mol | Decrease | Decrease stability | 0.949 |
| rs60864230 | R133Q | Probably damaging | Disease | Benign | Weakly destabilizing | 0.237 kcal/mol | Decrease | Decrease stability | 0.337 |
| [rs138592977](https://www.ncbi.nlm.nih.gov/snp/rs138592977) | R335L | Probably damaging | Disease | Pathogenic | Neutral | 0.186 kcal/mol | Decrease | a | 0.523 |
| rs59270054 | E82Q | a | Neutral | Pathogenic | Neutral | -0.035 kcal/mol | Decrease | Increase stability | 0.642 |
| rs28928903 | A57S | Probably damaging | Neutral | Pathogenic | Weakly destabilizing | 0.012 kcal/mol | Decrease | Decrease stability | 0.475 |
| rs60662302 | G602S | Probably damaging | Neutral | Benign | Neutral | -0.088 kcal/mol | Decrease | Increase stability | 0.055 |
| rs899373360 | I63V | a | Neutral | Pathogenic | Weakly destabilizing | -0.251 kcal/mol | Decrease | Decrease stability | 0.198 |
| rs267607571 | R190Q | Probably damaging | Disease | Pathogenic | Weakly destabilizing | -0.194 kcal/mol | Decrease | Decrease stability | 0.743 |
| [rs878855231](https://www.ncbi.nlm.nih.gov/snp/rs878855231) | D364N | Probably damaging | Neutral | Pathogenic | Weakly destabilizing | -0.141 kcal/mol | Increase | Decrease stability | 0.565 |
| [rs57966821](https://www.ncbi.nlm.nih.gov/snp/rs57966821) | E33D | Probably damaging | Disease | Pathogenic | Neutral | 0.128 kcal/mol | Decrease | Decrease stability | 0.452 |
| [rs58912633](https://www.ncbi.nlm.nih.gov/snp/rs58912633) | S143F | Probably damaging | Disease | Pathogenic | Weakly destabilizing | 0.649 kcal/mol | Increase | Decrease stability | 0.572 |
| [rs747998566](https://www.ncbi.nlm.nih.gov/snp/rs747998566) | L137V | Probably damaging | Disease | Benign | Destabilizing | -0.102 kcal/mol | Decrease | Decrease stability | 0.495 |
| [rs61282106](https://www.ncbi.nlm.nih.gov/snp/rs61282106) | G465D | Probably damaging | Disease | Pathogenic | Weakly destabilizing | -0.604 kcal/mol | Decrease | Decrease stability | 0.694 |
| [rs1222398892](https://www.ncbi.nlm.nih.gov/snp/rs1222398892) | K122Q | a | Disease | Pathogenic | Weakly destabilizing | -0.095 kcal/mol | Increase | Decrease stability | 0.523 |
| [rs58327533](https://www.ncbi.nlm.nih.gov/snp/rs58327533) | R25C | a | Disease | Pathogenic | Weakly destabilizing | -0.287 kcal/mol | Increase | Decrease stability | 0.655 |
| rs899373360 | I63L | Probably damaging | Disease | Pathogenic | Weakly destabilizing | -0.095 kcal/mol | Decrease | Decrease stability | a |

***^a:^*** *No prediction result could be obtained for this variant using the corresponding tool*
