# Supplementary material for: Computational Characterization of Pathogenic LMNA Missense Variants: Structural Instability, Altered Binding, and Conformational Dynamics
Source: Hum Mutat. 2026 Aug 26;2026:2444803. doi: 10.1155/humu/2444803 (PMC13508025; doi:10.1155/humu/2444803)
Supplement: Supplementary file 3 [file HUMU-2026-2444803-s003.docx]

**Supplementary Table S2**. LMNA variants, Evo2 delta scores, and associated disease annotations.

| rsID | Evo2 delta score | Related disease |
| --- | --- | --- |
| rs754097769 | -0.584676838 | Cardiovascular phenotype (Uncertain significance) |
| rs794728609 | -0.569397273 | * |
| rs2102889960 | -0.569293723 | Charcot-Marie-Tooth disease type 2 (Pathogenic) |
| rs1553265180 | -0.001407316 | Dilated cardiomyopathy 1A (Uncertain significance) |
| rs60864230 | -0.001164725 | LMNA-related disorder; Charcot-Marie-Tooth disease type 2 |
| rs138592977 | -0.001164725 | Dehydrated hereditary stomatocytosis 2 |
| rs267607582 | -0.000111074 | * |
| rs878855231 | -0.001091894 | Charcot-Marie-Tooth disease type 2 |
| rs57966821 | -0.001054347 | Charcot-Marie-Tooth disease type 2 |
| rs58912633 | -0.001042782 | Congenital muscular dystrophy due to LMNA mutation |
| rs59270054 | -0.001036042 | Cardiovascular phenotype |
| rs2102817667 | -0.001035177 | Charcot-Marie-Tooth disease type 2 |
| rs747998566 | -0.001020310 | Emery-Dreifuss muscular dystrophy 2 |
| rs61282106 | -0.000993508 | Familial partial lipodystrophy |
| rs59270054 | -0.000970455 | Charcot-Marie-Tooth disease type 2 |
| rs2102886618 | -0.000926193 | Charcot-Marie-Tooth disease type 2 |
| rs59931416 | -0.000903730 | -- |
| rs371635492 | -0.000901995 | Benign |
| rs57966821 | -0.000088115 | Charcot-Marie-Tooth disease type 2 |
| rs2102817667 | -0.000866438 | Charcot-Marie-Tooth disease type 2 |
| rs137969290 | -0.000863840 | Benign |
| rs1222398892 | -0.000859849 | Charcot-Marie-Tooth disease type 2 |
| rs58327533 | -0.000852875 | Cardiovascular phenotype; Charcot-Marie-Tooth disease type 2 |
| rs2102889960 | -0.000825854 | Cardiovascular phenotype |
| rs58922911 | -0.000818202 | Charcot-Marie-Tooth disease type 2 |
| rs886043109 | -0.000815442 | Charcot-Marie-Tooth disease type 2 |
| rs59931416 | -0.000759715 | Charcot-Marie-Tooth disease type 2 |
| rs28928903 | -0.000746164 | Dilated cardiomyopathy-hypergonadotropic hypogonadism syndrome |
| rs2102886618 | -0.000740563 | Charcot-Marie-Tooth disease type 2 |
| rs397517909 | -0.000740436 | Charcot-Marie-Tooth disease type 2 |
| rs60864230 | -0.000717107 | Familial partial lipodystrophy; Charcot-Marie-Tooth disease type 2; Hutchinson-Gilford progeria syndrome |
| rs28928903 | -0.000682314 | Dilated cardiomyopathy-hypergonadotropic hypogonadism syndrome |
| rs2102818172 | -0.000680648 | Charcot-Marie-Tooth disease type 2 |
| rs1553265180 | -0.000667801 | Dilated cardiomyopathy 1A |
| rs28928903 | -0.000665574 | Dilated cardiomyopathy-hypergonadotropic hypogonadism syndrome |
| rs1572358860 | -0.000648853 | Charcot-Marie-Tooth disease type 2 |
| rs2527973813 | -0.000642094 | * |
| rs60458016 | -0.000641983 | * |
| rs60662302 | -0.000627712 | Insulin-resistant diabetes mellitus; Congenital muscular dystrophy |
| rs762718963 | -0.000625109 | Benign |
| rs60992550 | -0.000058235 | Cardiovascular phenotype; Charcot-Marie-Tooth disease type 2 |
| rs899373360 | -0.000578241 | Charcot-Marie-Tooth disease type 2 |
| rs2527989011 | -0.000576753 | Charcot-Marie-Tooth disease type 2 |
| rs267607599 | -0.000560977 | Emery-Dreifuss muscular dystrophy 2; Charcot-Marie-Tooth disease type 2 |
| rs267607561 | -0.000560182 | Cardiovascular phenotype; Charcot-Marie-Tooth disease type 2 |
| rs56816490 | -0.000554133 | Charcot-Marie-Tooth disease type 2; Cardiovascular phenotype; LMNA-related disorder |
| rs2527833277 | -0.000549460 | E82G Charcot-Marie-Tooth disease type 2 |
| rs749784223 | -0.000536257 |  |
| rs1651352665 | -0.000528427 | Cardiovascular phenotype; Charcot-Marie-Tooth disease type 2 |
| rs267607571 | -0.000515329 | Cardiovascular phenotype; Charcot-Marie-Tooth disease type 2 |
