# Supplementary material for: Computational Characterization of Pathogenic LMNA Missense Variants: Structural Instability, Altered Binding, and Conformational Dynamics
Source: Hum Mutat. 2026 Aug 26;2026:2444803. doi: 10.1155/humu/2444803 (PMC13508025; doi:10.1155/humu/2444803)
Supplement: Supplementary file 4 [file HUMU-2026-2444803-s004.docx]

**Supplementary Table S1**. Pathogenicity and stability predictions for selected LMNA missense variants using multiple computational prediction tools.

| SNP | Mutation | PANTHER | PhD-SNP | E-SNPs&GO | INPS-MD | DynaMut | I-Mutant2.0 | MUpro | MutPred | Associated disease |
| --- | --- | --- | --- | --- | --- | --- | --- | --- | --- | --- |
| rs79907212 | D300G | Possibly damaging | Neutral | 0.93 | Weakly destabilizing | -0.517 kcal/mol (Destabilizing) | Decrease | Decrease stability | a | Hutchinson-Gilford progeria syndrome, atypical |
| rs60652225 | L140R | Possibly damaging | Disease | 0.93 | Neutral | 1.806 kcal/mol (Stabilizing) | Decrease | Decrease stability | a | Hutchinson-Gilford progeria syndrome, childhood-onset |
| rs60310264 | E145K | Possibly damaging | Disease | 0.93 | Neutral | 0.150 kcal/mol (Stabilizing) | Increase | Decrease stability | a | Hutchinson-Gilford progeria syndrome, atypical |
| rs267607547^b^ | M540T^b^ | Possibly damaging | Neutral | Pathogenic | Destabilizing | -0.603 kcal/mol | Decrease | Decrease stability | a | The only M540T variant located in the globular region |

***^a:^*** *No prediction result could be obtained for this variant using the corresponding tool.*

***^b:^****M540T (rs267607547) is included as a positive control — a known pathogenic variant localized within the globular Ig-fold domain — rather than an Evo2-prioritized candidate.*
