# Supplementary material for: Computational Characterization of Pathogenic LMNA Missense Variants: Structural Instability, Altered Binding, and Conformational Dynamics
Source: Hum Mutat. 2026 Aug 26;2026:2444803. doi: 10.1155/humu/2444803 (PMC13508025; doi:10.1155/humu/2444803)
Supplement: Supplementary file 5 [file HUMU-2026-2444803-s005.docx]

**Supplementary Table S5**. Comparative analysis of physicochemical changes and predicted structural effects induced by selected LMNA missense variants based on in silico evaluation.

| Variant | Wild-Type Amino Acid | | | Mutant-Type Amino Acid | | |
| --- | --- | --- | --- | --- | --- | --- |
|  | Size | Charge | Hydrophobicity | Charge | Mutant Residue | Damaging to the protein conservation |
| R133L | > | Positive | < | Neutral | loss of hydrogen bonds, disturb structure | Probably No |
| T157M | < | a | < | a | loss of hydrogen bonds, disturb structure | Probably Yes |
| T157K | < | Neutral | > | Positive | loss of hydrogen bonds, disturb structure | Probably No |
| T157R | < | Neutral | > | Positive | loss of hydrogen bonds, disturb structure | Probably Yes |
| L204Q | < | a | > | a | disturb interactions | Probably Yes |
| R190Q | > | Positive | a | Neutral | loss of external interactions, disturb the ionic interaction | Probably Yes |
| E82G | > | Negative | < | Neutral | loss of external interactions | Probably No |
| E317K | < | Negative | a | Positive | might lead to bumps | Probably Yes |
| N456D | a | Neutral | a | Negative | can lead to protein folding problems. | Probably Yes |
| N456T | > | a | < | a | loss of hydrogen bonds, disturb correct folding | Probably Yes |
| A57P | < | a | a | a | disturb interactions | Probably Yes |
| E65G | > | Negative | < | Neutral | loss of external interactions. | Probably No |
| L204R | < | Neutral | > | Positive | disturb interactions | Probably No |
| L245P | > | a | a | a | loss of external interactions. | Probably No |
| L59Q | < | a | > | a | loss of hydrophobic interaction | Probably No |
| R28Q | > | Positive | a | Neutral | loss of external interactions. | Probably Yes |
| R50G | > | Positive | < | Neutral | loss of external interactions. | Probably No |
| L313P | > | a | a | a | this might lead to loss of interactions. | Probably Yes |
| A43E | < | Neutral | > | Negative | loss of hydrophobic interactions | Probably Yes |
| R133Q | > | Positive | a | Neutral | loss of external interactions. | Probably No |
| R335L | > | Positive | < | Neutral | loss of hydrogen bonds and/or disturb correct folding. | Probably Yes |
| E82Q | a | Negative | a | Neutral | loss of interactions | Probably No |
| A57S | < | a | > | a | loss of hydrophobic interactions | Probably Yes |
| G602S | < | a | a | a | this might lead to bumps. | Probably No |
| I63V | > | a | a | a | loss of external interactions. | Probably No |
| R190Q | > | Positive | a | Neutral | loss of external interactions. | Probably No |
| D364N | a | Negative | a | Neutral | loss of interactions | Probably Yes |
| E33D | > | a | a | a | loss of external interactions. | Probably No |
| S143F | < | a | < | a | disturb interactions | Probably Yes |
| L137V | > | a | a | a | loss of external interactions. | Probably No |
| G465D | < | Neutral | > | Negative | lead to protein folding problems. | Probably Yes |
| K122Q | > | Positive | a | Neutral | loss of external interactions. | Probably No |
| R25C | > | Positive | < | Neutral | loss of hydrogen bonds and/or disturb correct folding. | Probably No |
